# Rehabilitation Needs Across Heterogenous Brazilian Regions: Secondary Analysis of the Global Burden of Disease Study

**DOI:** 10.3390/ijerph22040486

**Published:** 2025-03-25

**Authors:** Rogério Olmedija de Araújo, Tiótrefis Gomes Fernandes, Tiago Silva Jesus

**Affiliations:** 1 Department of Morphology, Institute of Biological Sciences, Federal University of Amazonas, Manaus 69067-005, AM, Brazil; 2 Faculty of Physiotherapy and Physical Education, Federal University of Amazonas, Manaus 69067-005, AM, Brazil; tiotrefis@ufam.edu.br; 3 Occupational Therapy Division, School of Health and Rehabilitation Sciences, The Ohio State University, Wexner Medical Center, Columbus, OH 43210, USA; tiago.jesus@osumc.edu

**Keywords:** disability, rehabilitation, global burden of disease, Brazil, health services needs and demand, health equity

## Abstract

**Aim:** This study aimed to determine the evolving rehabilitation needs in Brazil, considering five main impairment categories for nationwide health-service planning, stratified per age groups, as well as Brazilian regions with inequitable development. **Methods:** Secondary analysis of the Global Burden of Disease study (1990–2019), using Years Lived with Disability (YLD) rates for all ages and age-standardized metrics. The set of health conditions amenable to rehabilitation were selected and organized based on the five impairment types, derived from Brazil’s public-based Care Network for People with Disabilities. **Results:** A Brazil-wide 24% growth (1990–2019) in overall rehabilitation needs per capita (i.e., YLD rates per 100,000 population) was observed, in addition to a 6% negative growth for age-standardized YLD rates. “Physical” impairments accounted for 77% of the Brazilian rehabilitation needs in 2019; 69% of these impairments come from musculoskeletal conditions. Rehabilitation needs growth was also observed across the five Brazilian regions, ranging from 16% to 25%. **Conclusions:** Rehabilitation needs are growing across Brazil and its regions as a result of population ageing and epidemiological transition. Brazilian regions with lower income and lower population density (e.g., with more rural or remote populations) also experienced growth in rehabilitation needs, even though these regions are often underserved by rehabilitation professionals.

## 1. Introduction

Population ageing, increased life expectancy, and the rising prevalence of non-communicable diseases have significantly contributed to the growing burden of disability worldwide and more so in lower-middle-income countries and emergent economies [1,2]. Worldwide analyses of data from the Global Burden of Disease study have estimated that about 2.4 billion people worldwide have a health condition that would benefit from rehabilitation [1]. Those worldwide rehabilitation needs have been growing between 66 to 69% since 1990 in absolute values (i.e., accounting for population growth) but also per capita: by 17% since 1990 [2,3]. More importantly, upper-middle-income countries have been the income-level group with the greatest increase in rehabilitation needs per capita (29.9%), about twice as much of the growth observed in either lower-middle-income or high-income nations: 17.7 and 15.6%, respectively [2].

A growing burden of disability requires the strengthening of rehabilitation services in health systems, especially where the supply is lower and the growth in needs is more pronounced [4,5,6,7]. Aligned with the International Classification of Functioning, Disability, and Health, rehabilitation entails interventions aimed at optimizing the functionality of individuals with health conditions that originate functional impairments, reducing their disability in interaction with the environment [4,5]. Hence, rehabilitation services enable people with disabilities to live, work, learn, and fully participate as members of their society [4,5,8,9]. Rehabilitation services can target a range of disabilities, from transitional to long-term, and across the lifespan [1,10,11], being increasingly considered an essential health service to be part of universal health coverage [12].

Brazil is a large upper-middle income country, home to 203 million people, and according to the World Bank, with GDP per capita of US$9032 in 2023, with this GDP having a yearly percent growth that ranged from −4% to 12% from 1990 to 2023. Brazil also has a large landmass of 8.5 million km^2^. Brazil is comprised the union (federal government), 27 states—part of 5 larger regions—and over 5500 municipalities. While Brazil is highly diverse, systemic inequalities persist—including territorial—that limit the opportunities of many individuals who live in poverty and with poor health and disability [13]. Subsequently, regional disparities exist in health indicators [14]. These disparities also exist in the distribution of rehabilitation resources such as the rehabilitation workforce. For instance, the Southwestern region (e.g., encompassing large metropolitan areas such as Sao Paulo, Rio de Janeiro, and Minas Gerais) had the highest densities in all rehabilitation professions in 2020, contrasting with the North, Northeast, and Central West, which have lower population density and income level [15].

Geographic inequities in accessing rehabilitation resources are not exclusive to Brazil or developing nations. Even in high-income nations, individuals with disabilities living in the least developed regions or more rural or remote areas have poorer access to rehabilitation resources [16,17] among other geographic-based disability disparities [17,18]. Lower access to rehabilitation services puts people with disabilities at risk of not attaining the same level of health and functioning compared to those who can better access rehabilitation resources [17,19,20,21]. In Brazil, persistent inequalities have been identified in health-service resources, access, and utilization, experienced by the population with lower socio-economic status and those who live in regions with lower population density, especially in the North [22,23,24,25].

Overall, in low- and middle-income countries, a key factor undermining the integration of rehabilitation in the policy development agendas is the lack of a documented problem definition [26,27]. While in Brazil there is now up-to-date documentation of regional inequalities in the rehabilitation human resources [15], we have no cross-regional data on rehabilitation needs and their evolution over time to more fully access a supply–need disparity.

The data from the Global Burden of Disease (GBD) study have been used to determine rehabilitation needs worldwide and across countries [1,2]. However, these data were neither used before to map the population rehabilitation needs within Brazil nor aligned with the Brazilian’s public-based Care Network for People with Disabilities (CNPD) [28]. Specifically, the CNPD frames the services for people with disabilities according to five impairment types: physical, auditory, visual, intellectual, and multiple impairments [28]. In this context, here we aim to use data from the GBD study to:

1. Determine Brazil’s global burden of disability amenable to rehabilitation, per capita and adjusted for population ageing, their evolution since 1990, and the distribution by the five pre-established impairment categories.
2. Identify how these values vary by (a) key age groups and (b) the five major Brazilian regions.

## 2. Materials and Methods

Design: This study refers to a country-specific and within-country secondary analysis of the GBD 2019, the largest meta-study of global epidemiological data previously used to determine rehabilitation needs worldwide [1,2].

**Figure 1:**
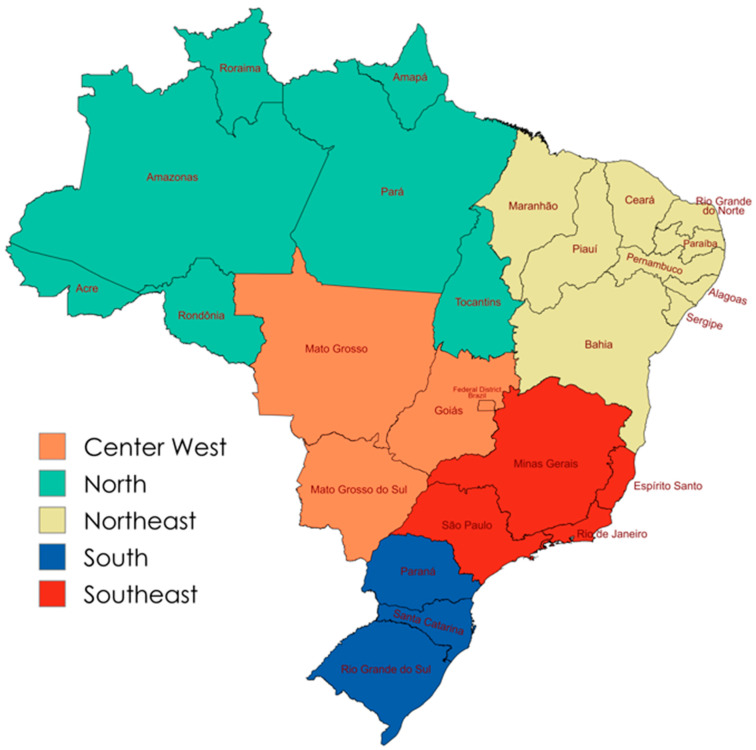
The five main Brazilian regions and their states. Created by the authors through Map Chart (https://www.mapchart.net/, accessed on 6 march 2025).

Setting: Our analysis is focused on Brazil as a nation first and then across five main regions. Figure 1 provides a map of the five Brazilian regions and the respective states within those regions. About 40% of the Brazilian population lives in Sao Paulo, Minas Gerais, and Rio de Janeiro, all part of the Southeast region, which, in total, has 91.8 inhabitants per km^2^. Conversely, the Center-West contains the lowest percentage (8%) of the population and the North has the lowest density (4.5 inhabitants per km^2^ and over 45% of the landmass) [13,29].

In both the North and Northeast, 30–40% of the population live below the poverty line, nearly twice as much as in other regions and with average income levels of about half of the three other regions [13,29].

**Health Conditions**: While informed by previous usages of the GBD data for rehabilitation needs assessments [1,2], the health conditions from which to extract data were adapted to reflect the five impairment categories of the Brazilian CNPD. The largest category of impairments (i.e., “physical”) was subdivided into four subcategories: musculoskeletal, neurological, cardiothoracic, and other physical health conditions. In turn, “multiple impairments”, here referring to early-onset conditions that can result in various impairment types, were also subdivided into congenital (i.e., present at birth) and neonatal (i.e., occurring during the first days of life) subcategories. Table 1 provides a detailed structure of the health conditions in the GBD from which we extracted data for Brazil and its regions.

Among the GBD measures, we extracted data only for Years Lived with Disability (*YLDs*) for each health condition in Table 1. The YLDs provide the overall, non-fatal impact of a health condition and consist of the number of years lived with any short-term or long-term sequelae of a health condition. Importantly, YLDs are also weighted for severity and disability; using stroke as an example, their disability weights range from 0.019 for mild consequences to 0.588 for severe consequences that include cognition impairments [30]. Because rehabilitation conditions with greater severity and disability may require more intensive rehabilitation services, YLDs can be a better indicator of rehabilitation needs than, for example, its underlying prevalence values. For the YLD measure, we extracted data for the “*rate*” (per 100,000 people) metric. For the “age”, we extracted YLDs for “*all ages*” and “*age-standardized*” rates in addition to three stratified age groups: 0–9 years, 10–54, and 55+. All that data extraction was performed for Brazil overall and for each of its five main regions, for both 1990 and 2019. Finally, we extracted data for the “total percentage change” of the YLD values for the year range of 1990–2021.

### Data Computation and Synthesis

With the data above extracted to Excel spreadsheets for each health condition in Table 1, we computed the summed estimates for each subcategory and the main category in Table 1. That applied to both 1990 and 2019 and to each of the six geographies addressed. Also, with the extracted measures and metrics, we were able to create two variables: YLD rates and age-standardized YLD rates: the former being the primary indicator of the rehabilitation need and the latter used to interpret any growth over time as being the result of (i.e., adjusted for) the population ageing. Percentage changes for (sub)categories of health conditions were computed using the summed estimates for both 2019 and 1990. The Brazil-wide results were tabulated using the structure of Table 1. Finally, we developed bar charts for displaying the distribution of the (a) YLD rates in Brazil per three main age groups and (b) YLD rates per the five main regions of Brazil for total values as well as values stratified per each of the five main health conditions.

## 3. Results

In our results, we respond to each question of our study.

- Brazil-wide rehabilitation needs, including those stratified per main condition groups

Table 2 shows that the primary indicator of rehabilitation needs (YLD rates, all ages) has increased by 24% from 1990 to 2019, with over 77% of these YLD rates in 2019 (i.e., 3847 out of 4986) coming from “physical” impairments alone. In turn, 69% of the “physical” impairments in 2019 (i.e., 2657 out of 3847 YLD rates) came from musculoskeletal conditions. In turn, the groups of conditions that had important percentage improvements since 1990 were neonatal, general musculoskeletal and pain conditions, and cardiac conditions.

Among individual conditions (see Table S1), some conditions associated with ageing, such as Alzheimer’s disease and other dementias, Parkison’s disease, and hypertensive heart disease, were among those that more than doubled their YLD rates from 1990 to 2019 in Brazil. Not surprisingly, Supplementary Table S1 shows a negative growth in the YLD rates for communicable neurological conditions and pulmonary conditions—many of them (e.g., tuberculosis) are communicable too.

Table 2 also presents the findings for age-standardized YLD rates (i.e., discounting the population ageing), which show frequent negative growth, emphasizing that the growth in YLD rates (i.e., per 100,000 population) has been essentially driven by population ageing.

(a) Distribution by age groups

**Figure 2:**
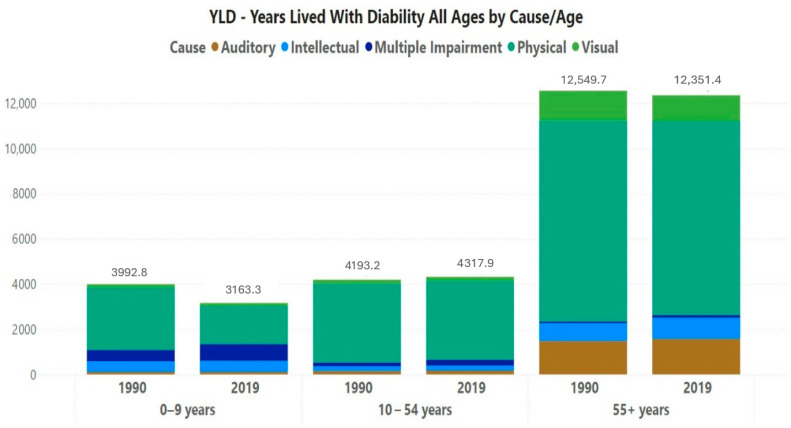
Brazil-wide YLD rates (all ages) as distributed by three major age groups.

Figure 2 provides the YLD rates (all ages) for the whole of Brazil distributed by three age groups, including stratified per impairment type and for both 1990 and 2019. In 2019, most YLDs, 62%, came from the population over 55 years, with values close to the numbers for 1990 and 2019 in the population 55+ years as well as 10–54 years. On the other hand, there was a decrease in YLD rates (3992.78 to 3163.32; −21%) in the 0–9 age group.

(b) Distribution by regions

**Figure 3:**
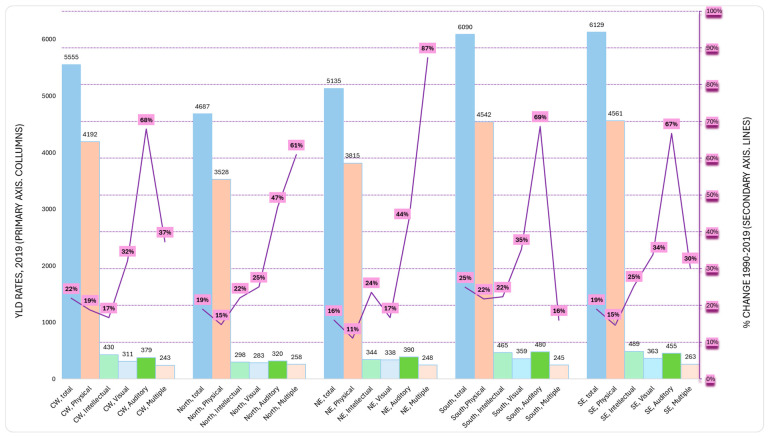
Brazilian YLD rates for all ages, distributed per its five main regions and impairment types, for the 2019 values (primary axis) and the percent change, 1990–2019 (secondary axis). Legend: CW: Central West, NE: Northeast, SE, Southeast.

Figure 3 provides the distribution of the Brazilian YLD rates per its five main regions and impairment types, including the 2019 values (primary axis) and the percent change from 1990 to 2019 (secondary axis). Most strikingly, percent growth in the YLD rates occurred in all five regions for all condition types, ranging from 11% for “physical” impairments to 87% for “multiple impairments”, both in the Northeast. Overall, the growth in “multiple impairments” was much higher in both the Northeast and North than in other more developed regions (e.g., 30% and 16%, respectively, for the South and Southeast).

The North, underdeveloped and with low population density, experienced an overall 19% growth in YLD rates between 1990 and 2019, the same growth as the most developed and densely populated Southeast region, but out of lower values in 1990 (3923 vs. 5114 YLD rates). Per impairment type, the more developed South and Southeast vs. the North and Northeast region had a pattern of having higher YLD rates in 2019, especially for “physical”, “intellectual”, and “auditory” impairments.

## 4. Discussion

This study is the first to analyze the rehabilitation needs for Brazil, including across age groups and its five main regions, from 1990 to 2019 and as stratified by impairment types. The latter reflected the organization of the Brazilian’s CNPD toward informing health-service planning for people with disabilities within the public health system. The results, derived from the GBD (i.e., the largest global epidemiological study) showed a Brazil-wide 24% growth (1990–2019) in overall rehabilitation needs per capita (i.e., YLD rates), aligned with the global trend for upper-middle-income countries [1,2]. Importantly, the study also highlighted population ageing as a key driver of increased rehabilitation needs since growth rates for age-standardized YLD rates were often negative despite the large increases per capita. As the Brazilian population 65 or older is expected to more than double by 2050 [31], rehabilitation needs (i.e., YLD rates) might grow even further in the future.

Historically, in Brazil, premature deaths resulting in lower life expectancy have been a greater issue in the North and Northeast regions than in the South and Southeast [14]. That may be part of the reason why YLD rates here were lower in both 1990 and 2019 for the North and Northeast. Yet, the growing trend has been similar across regions. Although with a partial time lag across regions, our data corroborate the notion that Brazil, as a whole, has undergone a so-called epidemiological transition; this comes from an increasing life expectancy, largely attributable to declines in communicable diseases occurring alongside an increased burden of non-communicable and chronic diseases [14]. As non-communicable diseases have been associated with YLDs [2,32], it was not surprising to find here that rehabilitation needs per capita were growing across all of Brazil’s regions.

The category of physical impairments was by far the one contributing more to the overall rehabilitation needs, Brazil-wide or stratified by either regions or age groups. Analyses of rehabilitation needs stratified by condition or impairment type are important for health systems and service planners, who need to allocate resources to different service types [5]. Although some rehabilitation services can be more “horizontal”, i.e., serving a range of rehabilitation needs across impairments, especially in the home, community-based or primary care, there are also care levels (e.g., inpatient rehabilitation) that target more specific populations and impairments [33]. Our data can be especially relevant for that level of planning, especially as the data are stratified per impairment types—which reflect the Brazilian’s CNPD [28]. Regarding human resources, for example, physical therapists are more likely to be necessary for addressing physical versus intellectual impairments; hence, our results are informative for that level of planning too, especially when combined with workforce supply data for Brazil as a whole or across its regions.

With regards to the latter, a recent study from Sixel et al. found that the density of rehabilitation professionals working for the public-based Unified Health System has been either declining or stabilizing in more recent years, likely because of some recent changes in policy and funding [15]. Those policies may impede the rehabilitation services and workforce to keep up with the growing population’s needs for rehabilitation, as shown in our study. Moreover, Sixel et al. found that the regions with a lower density of rehabilitation professionals were typically in the North or Northeast; these findings do not account for professionals working in private practice or with private and insurance-based reimbursement, likely more prevalent in the South and Southeast [15] As the North and Northeast regions have lower income and rates of private insurance [22], one can hypothesize that the regional disparity in the total rehabilitation workforce supply is much larger than that for the Unified Health System alone. That can be a matter of equity concern as the rehabilitation in the North and Northeast areas is also growing, often at similar levels to other Brazilian regions, as shown here. Furthermore, rehabilitation needs in the North and Northeast regions are expected to grow further as part of an epidemiological transition that has been occurring Brazil-wide [14]. For instance, as life expectancy increases in both the North and Northeast as a result of life-saving healthcare advances, the rates of physical, intellectual/cognitive, and auditory impairments—all of them highly associated with population ageing [32]—are likely to approach those of the more developed Brazilian regions.

The case of Brazil’s North region can be especially complex to handle for the equitable population access to needed rehabilitation resources. In addition to having low-income indicators, the region’s landmass is substantive, including the Amazon rainforest, while the population density is the lowest among Brazil’s main regions [13,29]. That occurs against the backdrop of underdeveloped infrastructures for both transportation and internet connectivity. All these characteristics are ecological risks that are known to negatively affect the access to rehabilitation services for the populations in need [34,35,36]. Known solutions to provide rehabilitation care to rural and remote populations, especially in high-income nations (e.g., telerehabilitation, outreach programs, or mobile units) [37,38,39,40] may be more complex to implement in the less developed and less densely populated areas of the North of Brazil, with its unique socio-demographic, socio-economic, and infrastructure profile (e.g., suboptimal roads or transportation facilities). Rehabilitation services integrated into local primary care services can be one solution [41], but it requires workforce availability, recruitment, and retention in those remote areas, which is also known to be complex [35,36].

All accounted for, there is a need for an integrated, purposeful approach and explicit public policies to facilitate the growing rehabilitation needs Brazil-wide, especially those in underserviced and remote locations, which need equity-oriented responses. Failure to do so can exacerbate the known and vicious cycle of poverty and disability, especially in lower-income contexts [42].

Finally, a lack of equitable access to rehabilitation may also contribute to an increase in the burden of disability in Brazil, especially in its underserviced regions. For example, a preventable or transitional disability arising from an acute or chronic condition can be transformed into a long-term, chronic, and more severe health condition or disability by a lack of access to quality rehabilitation treatment, either timely or at all [43]. In that sense, future research might investigate whether greater or lower access to rehabilitation resources across Brazilian regions affects their comparative burden of disability.

### Limitations

This study has several limitations. First, it uses YLD rates as a proxy estimator of rehabilitation needs; while this is likely the best proximal indicator from the GBD study and is often used to estimate large-scale rehabilitation needs [1,2], it is not the theoretical ideal functional-based indicator of rehabilitation needs. Second, this study uses data from the GBD 2019 (extraction occurred in late 2023), while the subsequent iteration (GBD 2021) was launched in 2024; hence, we only provide data until 2019. However, previous analyses have shown that overall rehabilitation needs have grown linearly over time and their growth pattern has not significantly changed with the use of an updated cycle [2,3]. Third, we did not analyze the multiple data points between 1990 and 2019, including linear regressions, partly because of the previously identified linear growth trends worldwide and in upper-middle-income countries [1]. Finally, we analyze the rehabilitation needs across five Brazilian regions, not as much at the state level, for a more parsimonious initial approach. Subsequent studies, including those matching rehabilitation needs with supply, may be more granular (e.g., at the state level).

## 5. Conclusions

Rehabilitation needs are growing Brazil-wide and across its regions, especially as a result of the increased life expectancy, population ageing, and epidemiological transition that is expected to have continued or accelerated growth in the upcoming decades. An explicit, data-based, needs-focused, equity-oriented health systems and human-resources policy and planning might be developed to address the growing burden of disability across Brazil. The data identified here, especially when combined with workforce data, can be instrumental for that. Finally, Brazil’s regions whose (remote) populations can experience greater risks of not accessing needed rehabilitation care, such as in the North region, may require regional-specific planning and both workforce strengthening and service delivery alternatives to ensure their growing rehabilitation needs are equitably met.

## Figures and Tables

**Table 1:** A detailed structure of the health conditions.

**Physical**	Musculoskeletal	General musculoskeletal	Osteoarthritis
			Rheumatoid arthritis
			Gout
			Other musculoskeletal disorders
		Traumatic musculoskeletal	Nature-based injuries—all listed except * and except **
		Musculoskeletal pain	Lower back pain
			Neck pain
	Neurological	Non-communicable neurological	Tension-type headache
			Stroke (cerebrovascular accident—CVA)
			Multiple sclerosis
			Parkinson’s disease
			Motor neuron disease
			Idiopathic epilepsy
			Other neurological disorders
			Epilepsy—except treated (impairment)
		Infectious neurological (communicable)	Meningitis
			Encephalitis
			Tetanus
			Zika
			Leprosy
			Guillain-Barré syndrome (impairment)
		Traumatic neurological	Spinal cord injury (nature-based injury)
			Traumatic brain injury (nature-based injury)
			Drowning (nature-based injury)
			Asphyxiation (nature-based injury)
	Cardiothoracic	Cardiac	Cardiovascular disease—all listed except CVA
			Heart failure—except treated, for all health conditions not selected otherwise
			Chagas disease
		Pulmonary	Chronic respiratory disease
			Severe chest injury (nature-based injury)
			Tuberculosis
			Lower respiratory disease
	Other physical	Cancer	Cancer
		HIV/AIDS	HIV/AIDS
		Burns	Burns
**Intellectual**	Intellectual and dementias	Intellectual and dementias	Intellectual disability (mild, moderate, severe, profound)
			Alzheimer’s and other dementias
			Autism
**Visual (Only)**	Visual	Visual	Blindness and vision loss—except presbyopia (impairment)
**Auditory (Only)**	Auditory	Auditory	Hearing loss—all except mild (impairment)
**Multiple Impairments**	Congenital	Congenital	Other congenital defects
			Neural tube defects
			Cleft lip and palate
			Down syndrome
			Turner syndrome
			Klinefelter syndrome
			Other chromosomal abnormalities
	Neonatal	Neonatal disorders	Neonatal disorders

* These included other categories: spinal and brain injuries, head injuries, asphyxia, burns to the lower airways, drowning, eye injuries, foreign body in the ear, and severe chest injuries. ** Minor, imprecise, or unrelated conditions to rehabilitation: Contusion; Superficial injury; foreign body in the respiratory system, foreign body in the gastrointestinal and urogenital systems, poisoning requiring injury care, open wounds, internal bleeding in the abdomen; Effects of different environmental factors; complications after therapeutic procedures.

**Table 2:** Brazil-wide YLD rates for all ages (i.e., the primary indicator of a rehabilitation need) and its age-standardized YLD rates (i.e., discounting the population ageing) for overall rehabilitation needs and stratified by main categories and subcategories in Table 1. The percentage change was computed from 1990 to 2019. MSK: Musculoskeletal.

	YLD Rates, All Ages		YLD Rates, Age-Standardized	
Rehabilitation Need Type	2019	Percentage Change	2019	Percentage Change
Overall Rehabilitation Needs	*4986*	*24%*	*4667*	*−6%*
Physical—total	3847	20%	3545	−9%
*MSK total*	2657	31%	2401	−4%
MSK general, total	973	43%	863	−3%
MSK trauma total	430	8%	398	−19%
MSK pain total	1255	33%	1140	1%
*Neurological total*	513	−2%	484	−24%
Neurological disorders NC—total	343	−6%	330	−27%
Neurological infectious/ communicable—total	9	−48%	9	−51%
Neurological trauma—total	160	16%	145	−11%
*Cardiothoracic—total*	488	-3%	488	−14%
Cardiac—total	151	39%	143	−5%
Pulmonary—total	336	−15%	346	−18%
*Other physical—total*	189	27%	171	−7%
Intellectual: developmental and dementias—total	253	28%	253	2%
Visual (only)—total	330	29%	313	−18%
Auditory (only)—total	259	24%	245	−22%
Congenital and neonatal total	298	78%	312	102%
*Congenital—physical—total*	23	−25%	25	−12%
*Neonatal total*	275	102%	287	128%

MSK: Musculoskeletal; NC: Non-communicable.

## Data Availability

These data were derived from the following resources available in the public domain: (https://vizhub.healthdata.org/gbd-results/, accessed on 6 March 2025).

## Supplementary Materials

The following supporting information can be downloaded at: https://www.mdpi.com/article/10.3390/ijerph22040486/s1, Table S1: Year Lived with Disability (YLD) Rates, for all ages and age-standardized, for Brazil as a whole as stratified per category and sub-category (colored) down to health condition type (black). Confidence Intervals only applicable for specific health conditions (i.e., results not aggregated).
