# Supplementary material for: Rehabilitation Needs Across Heterogenous Brazilian Regions: Secondary Analysis of the Global Burden of Disease Study
Source: Int J Environ Res Public Health. 2025 Mar 25;22(4):486. doi: 10.3390/ijerph22040486 (PMC12027096; doi:10.3390/ijerph22040486)
Supplement: Supplementary file 1 [file ijerph-22-00486-s001.zip › ijerph-3403414-supplementary.pdf]

Type of Rehabilitation Needs	YLD Rates, All ages (CI)		YLD Rates, Age standardized (CI)	
	2019	Percentage change	2019	Percentage change
**Overall Rehabilitation Needs**	***4985.7***	***24%***	***4667.4***	***-6%***
**Physical total**	***3846.5***	***20%***	***3544.9***	***-9%***
**MSK Total**	***2657.2***	***31%***	***2401.3***	***-4%***
MSK GENERAL Total	***972.6***	***43%***	***863.4***	***-3%***
Gout	9 (5,7 to 13)	97% (96% to 98%)	8,1 (5,1 to 11,6)	22% (15% - 28%)
Osteoarthritis	229,6 (115,2 to 454,7)	100% (99% to 100%)	206,2 (103,6 to 407,8)	10% (7% - 14%)
Rheumatoid Arthritis	40,9 (27,8 to 54,6)	51% (42% to 61%)	36,3 (24,7 to 48,3)	10% (6% - 13%)
Other musculoskeletal disorders	693 (473,5 to 967,7)	29% (22% to 37%)	612,8 (420,1 to 856)	-8% (-5% - -11%)
MSK Trauma Total	429.88	8%	***398.4***	***-19%***
Dislocation of shoulder	0,8 (0,2 to 1,5)	-14% (-9% to -18%)	0,7 (0,2 to 1,5)	-13% (-9% to -18%)
Dislocation of knee	1,9 (1,2 to 2,9)	5% (3% to 6%)	1,8 (1,2 to 2,8)	-14% (-10% to -19%)
Dislocation of hip	2,6 (1,3 to 4,4)	26% (19% to 33%)	2,4 (1,2 to 4)	-14% (-9% to -18%)
Fractures	279,1 (189,8 to 397,2)	19% (13% to 24%)	255,2 (173,4 to 362,2)	-15% (-10% to -20%)
Crush injury	5,9 (4,1 to 8,3)	29% (22% to 37%)	5,4 (3,8 to 7,6)	-12% (-8% to -16%)
Amputations	107,2 (72,8 to 156,8)	-7% (-4% to -9%)	97,9 (66,4 to 143,1)	-31% (-23% to -39%)
congenital musculoskeletal and limb anomalies	32,4 (20,4 to 47)	-13% (-8% to -17%)	34,9 (21,8 to 51)	3% (2% to 4%)
MSK Pain Total	***1254.7***	***33%***	***1139.6***	***1%***
Low back pain	943,4 (665,5 to 1253,4)	32% (23% to 40%)	861,5 (607,2 to 1146,4)	1% (1% to 2%)
Neck pain	250,4 (164,7 to 365,7)	43% (33% to 52%)	221,7 (145,4 to 322,6)	0% (0% to 0%)
Tension-type headache	60,9 (17,1 to 220,6)	18% (12% to 23%)	56,4 (15,3 to 206,7)	0% (0% to 0%)
**Neurological Total**	***513.1***	***-2%***	***484.0***	***-24%***
Neurological disorders NC - Total	***343.5***	***-6%***	***329.5***	***-27%***
Stroke	130,9 (96,2 to 165,1)	11% (7% to 15%)	120,9 (88,7 to 152,6)	-33% (-25% to -41%)
Multiple sclerosis	5,7 (3,9 to 7,8)	71% (64% to 79%)	5 (3,4 to 6,9)	16% (11% to 21%)
Parkinson's disease	13,2 (9 to 18,2)	135% (126% to 143%)	12,5 (8,5 to 17,1)	20% (14% to 26%)
Idiopathic epilepsy	126,1 (72,1 to 194,1)	-26% (-18% to -33%)	125,5 (71,5 to 193,8)	-27% (-20% to -35%)
Epilepsy - except treated (impairment)	36,2 (22,1 to 55,9)	-2% (-1% to -2%)	33,1 (20,2 to 51)	-37% (-28% to -45%)
Motor neuron disease	0,6 (0,4 to 0,9)	26% (19% to 33%)	0,6 (0,4 to 0,8)	17% (12% to 22%)
Other neurological disorders	30,7 (19,1 to 45,3)	-3% (-2% to -5%)	31,9 (19,7 to 47)	4% (2% - 5%)
Neurological Infectious / Communicable - Total	***9.5***	***-48%***	***9.1***	***-51%***
Guillain Barre (impairment)	0,2 (0,1 to 0,3)	-24% (-17% to -31%)	0,2 (0,1 to 0,3)	-41% (-32% to -50%)
Meningitis	6,3 (4,5 to 8,3)	-50% (-41% to -59%)	6,1 (4,3 to 8)	-51% (-41% to -60%)
Encephalitis	1,8 (1,2 to 2,4)	-61% (-52% to -70%)	1,7 (1,2 to 2,2)	-64% (-56% to -73%)
Tetanus	0 (0 to 0)	-89% (-85% to -93%)	0 (0 to 0)	-90% (-86% to -93%)
Zika***	0 (0 to 0)	Not applicable	0 (0 to 0)	Not applicable
Leprosy	1,2 (0,7 to 1,7)	42% (33% to 52%)	1 (0,7 to 1,5)	-8% (-5% to -11%)
Neurological Trauma - Total	***160.2***	***16%***	***145.4***	***-11%***
Spinal Injuries	108,2 (77,5 to 137,9)	11% (7% to 14%)	98,4 (70,3 to 125,7)	-13% (-9% to -17%)
Head Injuries	50 (34,9 to 67,9)	29% (21% to 37%)	45,2 (31,6 to 61,3)	-6% (-4% to -8%)
Drowning and nonfatal submersion	2 (1,3 to 2,8)	24% (17% to 31%)	1,8 (1,2 to 2,5)	-14% (-9% to -18%)
Asphyxiation	0,1 (0 to 0,1)	-20% (-14% to -27%)	0,1 (0 to 0,1)	-18% (-13% to -24%)
**Cardiothoracic - Total**	***487.51***	***-3%***	***488.2***	***-14%***
Cardiac - Total	***151.1***	***39%***	***142.6***	***-5%***
Chagas disease	9,1 (5,8 to 13,1)	-3% (-2% to -3%)	8,2 (5,3 to 11,9)	-34% (-26% to -42%)

Mild heart failure	0,1 (0 to 0,1)	130% (122% to 138%)	0,1 (0 to 0,1)	30% (22% to 37%)
Moderate heart failure	0,1 (0 to 0,1)	130% (122% to 138%)	0,1 (0 to 0,1)	29% (22% to 37%)
Severe heart failure	0,4 (0,2 to 0,7)	129% (121% to 137%)	0,4 (0,2 to 0,7)	29% (21% to 37%)
Rheumatic heart disease	45,9 (27 to 71)	7% (4% to 9%)	43,5 (25,7 to 67,4)	2% (2% to 3%)
Ischemic heart disease	67,6 (44,7 to 97,1)	80% (74% to 86%)	62,7 (41,5 to 89,5)	-5% (-3% to -7%)
Hypertensive heart disease	14,4 (8,4 to 23,3)	112% (108% to 116%)	13,9 (8,2 to 22,6)	-1% (0% to -1%)
Cardiomyopathy and myocarditis	7,4 (4,6 to 11,2)	81% (75% to 87%)	7,1 (4,4 to 10,8)	-2% (-1% to -3%)
Non-rheumatic valvular heart disease	0,5 (0,3 to 0,8)	206% (204% to 208%)	0,4 (0,2 to 0,8)	63% (55% to 72%)
Congenital heart anomalies	5,7 (2,6 to 9,6)	-21% (-15% to -27%)	6,3 (2,9 to 10,6)	-5% (-4% to -7%)
Pulmonary - Total	***336.4***	***-15%***	***345.6***	***-18%***
Tuberculosis	11,7 (7,9 to 16,3)	-38% (-29% to -47%)	11 (7,4 to 15,3)	-46% (-36% to -55%)
lower respiratory infections	11,2 (7,5 to 15,7)	-10% (-6% to -13%)	10,9 (7,3 to 15,5)	-25% (-18% to -33%)
Chronic respiratory diseases	307,3 (219,2 to 416,7)	-14% (-9% to -18%)	317,5 (224,5 to 441,9)	-16% (-11% to -21%)
Severe chest injury	6,1 (3,7 to 9,4)	-10% (-6% to -13%)	5,9 (3,6 to 9,2)	-13% (-9% to -17%)
Lower airway burns	0,2 (0,1 to 0,3)	4% (2% to 5%)	0,2 (0,1 to 0,3)	-10% (-7% to -14%)
**Oher physical - total**	***188.6***	***27%***	***171.3***	***-7%***
Neoplasms	79,7 (58,3 to 102,7)	99,8% (99% to 99%)	72,9 (53,2 to 93,9)	22% (16% to 29%)
HIV/AIDS	42,9 (28,2 to 61,9)	217% (212% to 222%)	38,2 (25 to 55,6)	183% (178 to 189%)
Burns	66 (36,3 to 110,9)	-31% (-23% to -39%)	60,3 (33,2 to 101,1)	-46% (-36% to -55%)
**Intellectual: Developmental and Dementias - total**	***252.5***	***28%***	***253.3***	***2%***
Mild intellectual disability	15,7 (5,4 to 29,4)	-20% (-14% to -26%)	16,1 (5,5 to 30,3)	-12% (-8% to -17%)
Moderate intellectual disability	4,7 (2,7 to 7,6)	-30% (-22% to -37%)	4,8 (2,8 to 7,8)	-26% (-18% to -33%)
Profound intellectual disability	4,2 (1,4 to 8,2)	-20% (-14% to -26%)	4,2 (1,4 to 8,3)	-18% (-13% to -24%)
Severe intellectual disability	3,2 (1,6 to 5,8)	-14% (-9% to -18%)	3,3 (1,6 to 5,9)	-9% (-6% to -12%)
Autism	112,9 (76,8 to 158,5)	-4% (-3% to -6%)	114,5 (77,9 to 160,9)	1% (1% to 2%)
Alzheimer's disease and other dementias	111,8 (78,5 to 149,8)	156% (147% to 166%)	110,4 (77,5 to 147,5)	8% (6% to 11%)
**Visual (only) - total**	***329.5***	***29%***	***312.6***	***-18%***
Blindness	131,9 (86,5 to 191,6)	28% (20% to 35%)	124,4 (81,6 to 180,7)	-30% (-22% to -38%)
Moderate Vision loss	124,4 (75,3 to 193,8)	26% (18% to 33%)	118,8 (72 to 185,1)	-4% (-2% to -5%)
Severe vision loss	73,2 (48,6 to 105,2)	39% (30% to 48%)	69,4 (46 to 99,6)	-13% (-9% to -17%)
**Auditory (only) - total**	***259.4***	***24%***	***245.1***	***-22%***
Complete hearing loss	11,6 (7,2 to 17,8)	7% (4% to 9%)	11,1 (6,9 to 17)	-31% (-23% to -39%)
Moderate hearing loss	105,2 (60,7 to 171)	26% (19% to 34%)	97,1 (55,9 to 157,9)	-22% (-15% to -28%)
Moderately hearing loss	79,7 (50,3 to 116,8)	42% (33% to 51%)	75,6 (48,1 to 110,6)	-75% (-69% to -81%)
Profound hearing loss	34,7 (21,4 to 52,4)	-1% (-1% to -2%)	34,1 (21,2 to 51,5)	-18% (-12% to -23%)
Severe hearing loss	28,1 (17,5 to 41,3)	23% (16% to 29%)	27,2 (16,7 to 40)	-21% (-15% to -28%)
**Congenital & Neonatal Total**	***297.8***	***78%***	***311.6***	***102%***
Congenital - physical - total	***23.2***	***-25%***	***25.1***	***-12%***
Other congenital birth defects	13 (6,4 to 25,2)	-23% (-16% to -30%)	13,4 (6,8 to 25,8)	-18% (-12% to -24%)
Neural tube defects	1,8 (1,1 to 2,8)	-31% (-23% to -39%)	2,1 (1,3 to 3,1)	-8% (-5% to -11%)
Orofacial clefts	1,3 (0,8 to 1,8)	-21% (-15% to -27%)	1,3 (0,8 to 1,9)	-15% (-10% to -20%)
Down Syndrome	2,4 (1,5 to 3,6)	-26% (-19% to -34%)	2,6 (1,7 to 4,1)	-4% (-2% to -5%)
Turner Syndrome	0,2 (0,1 to 0,3)	-17% (-12% to -23%)	0,2 (0,1 to 0,3)	-3% (-2% to -4%)
Klinefelter Syndrome	0 (0 to 0)	0% (0% to 0%)	0 (0 to 0)	25% (18% to 32%)
Other chromosomal abnormalities	4,6 (3,1 to 6,6)	-27% (-19% to -34%)	5,5 (3,7 to 7,8)	1% (1% to 1%)
Neonatal total	***274.57***	***102%***	***286.5***	***128%***

Supplementary table S1: Year Lived with Disability (YLD) Rates, for all ages and age-standardized, for Brazil as a whole as stratified per category and sub-category (colored) down to health condition type (black). Confidence Intervals only applicable for specific health conditions (i.e. results not aggregated).
